# Human Chorionic Gonadotropin Influences Systemic Autoimmune Responses

**DOI:** 10.3389/fendo.2018.00742

**Published:** 2018-12-06

**Authors:** Alpana De, Ruchi Sachdeva, Anjali Bose, Monika Malik, Nipun Jayachandran, Rahul Pal

**Affiliations:** Immunoendocrinology Lab, National Institute of Immunology, New Delhi, India

**Keywords:** human chorionic gonadotropin, autoimmunity, autoreactivity, lupus, systemic lupus erythematosus

## Abstract

Immunopathological outcomes in Systemic Lupus Erythematosus (SLE; or lupus) are believed to be autoantibody-mediated. Conditions which promote a Th2 skew (such as pregnancy) should encourage antibody production, worsening antibody-mediated diseases while ameliorating Th1/Th17-mediated diseases. Although an increased propensity toward autoreactivity can be observed in pregnant lupus patients and in pregnant lupus-prone mice, whether a unique human pregnancy-specific factor can contribute to such effects is unknown. This study assessed whether human chorionic gonadotropin (hCG, a pregnancy-specific hormone of diverse function) at physiological concentrations could mediate stimulatory influences on immune parameters in non-pregnant, lupus-prone mice, in light of the hormone's ameliorating effects on Th1-mediated autoimmunity in murine models. Results demonstrate that administration of hCG heightened global autoreactivity in such mice; antibodies to dsDNA, RNP68, Protein S, Protein C, β2-glycoprotein 1, and several phospholipids were enhanced, and hormone administration had adverse effects on animal survival. Specifically in splenic cell cultures containing cells derived from lupus-prone mice, hCG demonstrated synergistic effects with TLR ligands (up-modulation of costimulatory markers on B cells) as well as with TCR stimuli (enhanced proliferative responses, enhanced levels of cytokines, and the phosphorylation of p38). In both instances, enhanced synthesis of lupus-associated cytokines was observed, in addition to the heightened generation of autoantibodies reactive toward apoptotic blebs. These results suggest that selective transducive, proliferative, and differentiative effects of hCG on adaptive immune cells may drive autoreactive responses in a lupus environment, and may also potentially provide insights into the association between the presence of higher hCG levels (or the administration of hCG) with the presence (or appearance) of humoral autoimmunity.

## Introduction

SLE is characterized by antibody responses against a large spectrum of self-antigens (1). Multiple factors, including sex, genetics, hormones, and environmental influences contribute to onset (2–5). Since SLE is primarily considered an autoantibody-mediated disease and represents an immune environment generally skewed toward a Th2 cytokine profile (6), other similarly physiologically and immunologically biased scenarios would be expected to perpetuate autoantibody production. Pregnancy is believed to constitute such an environment, where heightened hormonal levels may influence disease (7, 8). Pregnancy-associated flares have been reported in certain studies (9), and disease-promoting effects of estrogen and prolactin have been described (4). Conversely, pregnancy often results in an amelioration of the symptoms of cell-mediated (Th1-associated) autoimmune diseases (10).

The glycoprotein hormone human chorionic gonadotropin (hCG) plays a critical role in the sustenance of pregnancy (11). Apart from organs of the reproductive system, receptors for hCG are also present in extra-gonadal tissues including T cells (12), B cells (13), and macrophages (14), suggesting the hormone may have as yet un-discovered effects. Its roles in mediating feto-maternal immune tolerance (15, 16) and postulated immunosuppressive activity has prompted investigation of its effects in animal models of organ-specific (Th1-mediated) autoimmunity; administration of hCG ameliorates symptoms in spontaneous murine models of Type-1 diabetes (17) and Sjögren's Syndrome (18).

The effects of hCG on systemic autoimmune responses have not been investigated, although reports in humans are suggestive of influence. Higher levels of hCG have been reported in pregnant lupus patients (19), and the injection of hCG in women is associated with ovarian hyper-stimulation syndrome (OHSS) (20, 21), a condition which can characterized by thrombosis, which in turn has been linked to the presence of anti-phospholipid antibodies. In addition, increased levels of hCG have been associated with preeclampsia (22, 23) another thrombotic condition with possible autoimmune involvement (24–26).

In the current study, the immunobiological roles mediated by hCG in a lupus milieu were determined. The data indicate that the hormone elicits autoreactive responses in specifically in lupus-prone mice both *in vivo* and *in vitro*, events that correlate with differentially heightened cytokine, phenotypic and signaling responses. These findings may help elucidate the association of hCG with immune and physiological aberrance in conditions such lupus, OHSS and preeclamspsia.

## Materials and Methods

### Ethics

This study was carried out in accordance with the guidelines laid down by the Committee for the Purpose of Control and Supervision of Experiments on Animals of the Government of India. The protocol was approved by the Institutional Animal Ethics Committee of the National Institute of Immunology (IAEC Number: 223/10).

### Mice

Female inbred lupus-prone mice (NZM2410 and NZB x NZW (F1), referred to as NZM and B/W F1, respectively) and healthy mice (BALB/c, FVB) were obtained from The Jackson Laboratory. Animals were bred at the Small Animal Facility of the National Institute of Immunology, New Delhi. For some experiments, B/W F1 mice were subjected to ovariectomy at 6 weeks of age. Blood samples were collected from the retro-orbital vein under anesthesia.

### Effect of Pregnancy on Humoral Autoimmune Responses

B/W F1 and BALB/c mice (*n* = 6) were rendered pregnant at 2 months, 6 months, and at 8 months of age. Blood samples were collected pre-pregnancy, at mid-pregnancy (Day 12) and at late-pregnancy (Day 19). Antibodies in pooled sera were evaluated for reactivity toward cellular moieties by Western blot and confocal microscopy, as described below.

### hCG

Native hCG (Wyong Biologicals) was employed in these studies. Though recombinant hCG is known to exhibit biological activity on reproductive tissues, the extent of oligosaccharide branching can be distinct from that on native hCG. This being the first study to assess the differential effects of hCG on healthy and lupus-prone animals (and on immune cells derived from such animals), it was considered appropriate to employ native hCG so as to study responses upon exposure to as natural a ligand as possible. The hormone preparation, characterized for its physical (by SDS-PAGE and HPLC), immunological (by a hCGβ-specific radioimmunoassay and Western blot), and biological (by radioreceptor assay and Leydig cell bioassay) properties, was found not to contain significant amounts of free hCG subunits, and exhibited activity in the range of ~11,000–13,000 IU/mg against relevant hCG standards, equivalent to recombinant hCG when the contribution of carbohydrates is taken into account.

### Effect of hCG on Humoral Autoimmune Responses *in vivo*

BALB/c, FVB, NZM, and B/W F1 mice (*n* = 6) were administered three intra-peritoneal injections of hCG per week (100 IU per injection) in 200 μl PBS (as vehicle) from the age of 8 weeks to 32 weeks; control mice received PBS. The duration, concentration and schedule of treatment was decided on the basis of several considerations, the primary amongst them being the reported circulating levels of the hormone during human pregnancy and its expected circulatory half-life, on preliminary dosing experiments which assessed the effects of hCG on humoral immune responses vis-a-vis the natural occurrence of autoantibodies in lupus-prone mice, and on the natural mortality rates of lupus-prone mice in the colony. Blood samples were withdrawn at fortnightly intervals and serum antibodies assessed for autoreactivity in assays described below.

CCL131 (murine neuroblastoma) cells, permeabilized by brief incubation in chilled methanol containing 0.1% Triton X-100, were incubated with diluted sera for 60 min at 4°C followed by a similar incubation with secondary antibody (anti-mouse IgG + IgM-FITC, Jackson ImmunoResearch). Subsequent to flow cytometry, data was analyzed by WinMDi 2.9 software. For the preparation of lysate, cells were washed twice with PBS by centrifugation at 320 g at 4°C for 5 min and incubated with 80 μl RIPA buffer (2% (v/v) Triton-X100, 1% (w/v) sodium deoxycholate, 0.1% (w/v) SDS, 1 mM Tris base, 150 mM sodium chloride) containing a protease inhibitor cocktail (20 μl/ml) for 20 min on ice; cells were freeze-thawed twice in liquid nitrogen. Tubes were centrifuged at 16,000 g for 15 min at 4°C. Protein concentrations in cellular lysates were determined by BCA and 200 μg protein was loaded on preparative gels. Using diluted sera derived from hCG-treated and vehicle-treated mice, Western blots on lysates were carried out by standard protocols and reactive bands were visualized by enhanced chemiluminescence. For confocal microscopy, permeabilized CCL131 or HeLa (human cervical cancer) cells were “blocked” by incubation with 10% normal horse serum for 16 h at 4°C. Cells were then incubated with diluted sera for 1 h at 37°C, followed by a similar incubation with Alexafluor 488-conjugated goat anti-mouse antibodies (Jackson ImmunoResearch). Cells were visualized on a Zeiss LSM 510 Meta confocal microscope (Jena) with 63x/1.4 oil immersion. The laser lines used were 640 Argon 458/477/488/514/641 nm (for FITC), and a Chameleon ultra auto-tunable femtosecond laser with a tuning range 690–1050 643 nm (for DAPI). LSM5 software was used for image acquisition.

Calf thymus dsDNA (Sigma) was dissolved at 100 μg/ml in PBS. Protein S, Protein C, β-2 glycoprotein 1, La, and RNP68 (Haematologic Technologies, Aerotec Diagnostics) were dissolved in carbonate buffer, pH 9.5. Synthetic lipids (Avanti Polar Lipids Inc.) were reconstituted in choloform:methanol (1:3); cardiolipin was reconstituted in ethanol. Reactivity of antibodies in diluted sera to these moieties was assessed by standard ELISA protocols. Data was plotted as O.D. units (serum dilution × O.D.), selecting dilutions of equivalence.

Whether anti-hCG antibodies were elicited upon the administration of hCG to NZM and FVB mice was ascertained by radioimmunoassay. Briefly, dilutions of mice sera were incubated with ^125^I-hCG (10,000 cpm; 40–60 μCi/μg) and 4% v/v horse serum at 4°C for 48 h. 12.5% w/v polyethylene glycol (Sigma; Mw 8000) was then added in order to precipitate any antigen-antibody complexes, followed by centrifugation at 1,500 g at 4°C for 20 min. Radioactivity in the pellet was assessed in a γ-counter (Perkin Elmer).

### Effect of hCG on Humoral Autoimmune Responses *ex vivo*

3 × 10^5^ splenocytes derived from B/W F1 and BALB/c mice were individually dispensed into wells previously adsorbed with anti-CD3 antibodies (1 μg/ml). Medium containing hCG (10 IU/ml) was added and an incubation carried out for 48 h. 5 × 10^5^ splenocytes derived from either NZM or FVB mice were cultured in presence of hCG (10 IU/ml) alone or in combination with TLR ligands: ssRNA40 (10 μg/ml) or ODN1826 (20 μM). Incubations were carried out for 24 h. Supernatants from these cultures were assessed for the presence of antibodies reactive toward apoptotic blebs (isolated from cultures of CCL131 cells upon incubation with 0.5 μM staurosporin (Bio Basic) for 24 h by differential centrifugation) by Western blot.

### Effect of hCG and TLR Ligand Co-signaling on B Cell Phenotype, and on Cytokine Responses

5 × 10^5^ splenocytes derived from either NZM or FVB mice were cultured in presence of hCG (10 IU/ml) alone or in combination with TLR ligands: ssRNA40 (10 μg/ml) or ODN1826 (20 μM). After incubation for 24 h, the expression of MHC 1, MHC 2, CD80, CD86, CD83 or CD40 on CD19^+^ cells were assessed by flow cytometry (BD VERSE); data was analyzed by Flow Jo X software. Cytokines (IFN-γ, IL-5, IL-10, TGF-β, TNF-α, IL-6, and IL-8) in supernatants collected at 48 h were quantified by ELISA (eBiosciences).

### Effect of hCG and TCR Co-Signaling on Proliferative and Cytokine Responses, and on Signaling Intermediates

3 × 10^5^ splenocytes derived from B/W F1 and BALB/c mice were individually dispended into wells previously adsorbed with anti-CD3 antibodies (1 μg/ml). Medium containing hCG (10 IU/ml) was added and an incubation carried out for 48 h at 37°C. Cellular proliferation was measured by the uptake of ^3^H-thymidine. In parallel experiments, supernatants were collected at 48 h for the quantification of the cytokines indicated above.

Splenocytes derived from B/W F1 and BALB/c mice were dispensed either individually or at different ratios, with individual numbers ranging between 10^5^ and 3 × 10^5^ cells per well. Cells were incubated in medium or in medium containing hCG (10 IU/ml) for 72 h. Cellular proliferation was measured by the uptake of ^3^H-thymidine.

5 × 10^6^ splenocytes were dispensed onto wells previously adsorbed with of anti-CD3 antibodies (1 μg/ml). hCG (10 IU/ml) was then dispensed and an incubation carried out at 37°C. After 15 min (for the assessment of ERK1/2 phosphorylation) or 30 min (for the assessment of AKT and p38 phosphorylation), cell lysates were processed for Western blot. The following antibodies were employed: AKT, phospho-AKT (CST); p38, phospho-p38, ERK1/2, phospho-ERK1/2, β-actin (Santa Cruz). Reactive bands were visualized by enhanced chemiluminescence and band intensities quantified employing ImageJ software.

### Statistical Analysis

Either the Shapiro-Wilk test or the Kolomogorov-Smirnov test were applied (depending on sample size) to assess for normal distribution of data; all data demonstrated normal distribution. Statistical analysis was carried out by the Student's *t*-test (for comparison between two groups) and ANOVA (for comparison between more than two groups and for paired data). Kaplan–Meier analysis was employed to assess influence of hCG administration on animal survival.

## Results

### Effect of Pregnancy on Humoral Autoimmune Responses

**Figure 1:**
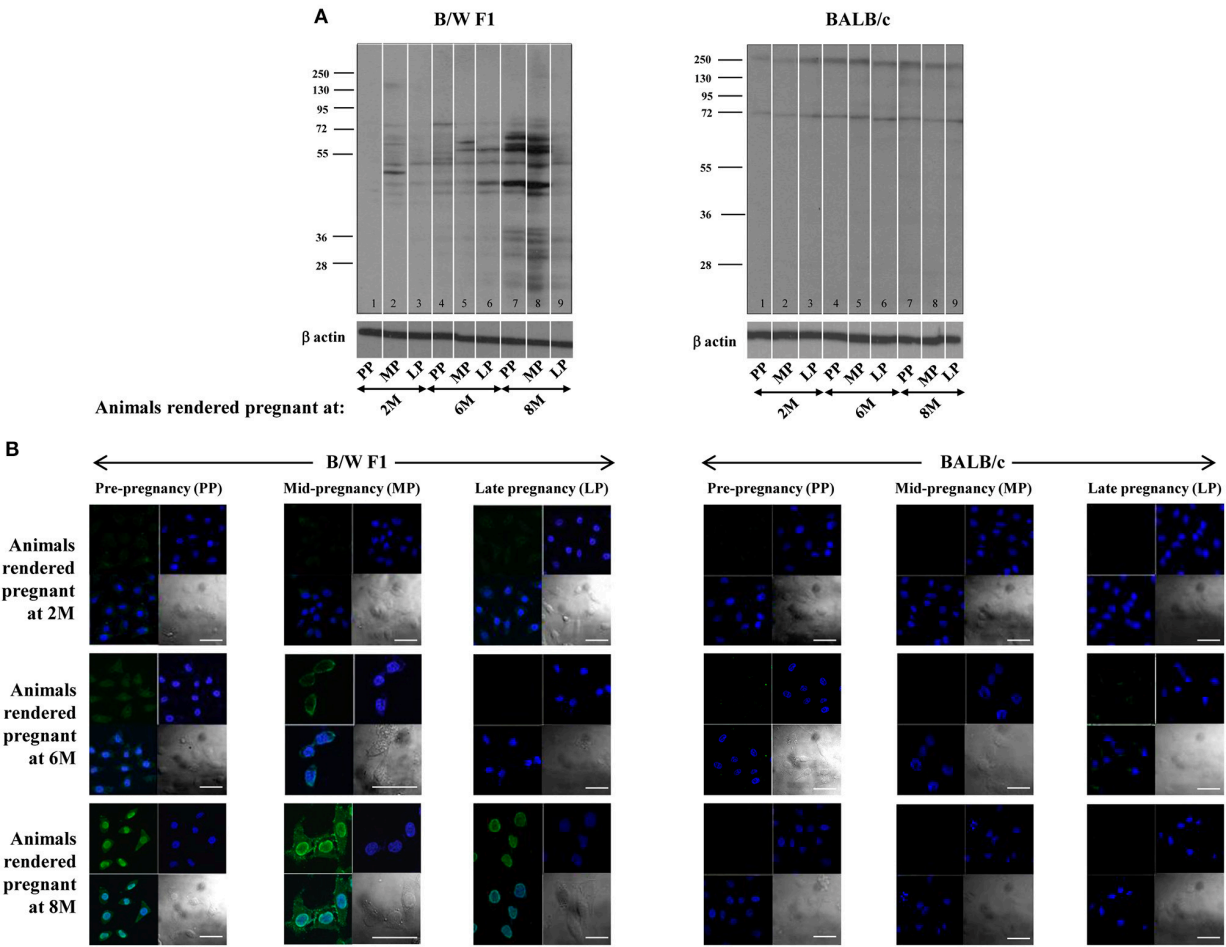
Effect of pregnancy on humoral autoimmune responses. B/W F1 and BALB/c mice were rendered pregnant at 2 months (2 M), 6 months (6 M), and 8 months (8 M) of age. **(A)** Western blot analysis of reactivity of antibodies in pooled sera [at pre-pregnancy (PP), mid-pregnancy (MP; Day 12), and late-pregnancy (LP; Day 19)] toward CCL131 cellular extracts. Anti-β actin antibodies were employed to verify equivalence of loading. **(B)** Confocal analysis. Reactivity toward HeLa cells of antibodies in pooled sera at pre-pregnancy (PP), mid-pregnancy (MP; Day 12), and late-pregnancy (LP; Day 19). Top right panels, DAPI; Top left panels, Antibody reactivity; Bottom left panel, Overlays; Bottom right panels, DIC. Bars: 20 μm.

Whether pregnancy preferentially affects the quantum and specificity of anti-self responses in a milieu of ongoing autoimmunity was assessed. In B/W F1 mice, anti-self reactivity was elevated as animals aged, as expected. Upon Western blot, heightened reactivity was observed of antibodies in sera derived mid-pregnancy which then declined at late-pregnancy, a pattern particularly discernible in lupus-prone mice rendered pregnant at 8 months (Figure 1A). Upon confocal analysis, such a pattern of reactivity was observable in such mice rendered pregnant at both 6 months and 8 months (Figure 1B). Such pregnancy-dependent modulation of humoral anti-self reactivity was not observed in BALB/c animals (Figures 1A,B).

### Effects of hCG on Humoral Autoimmune Responses *in vivo*

**Figure 2:**
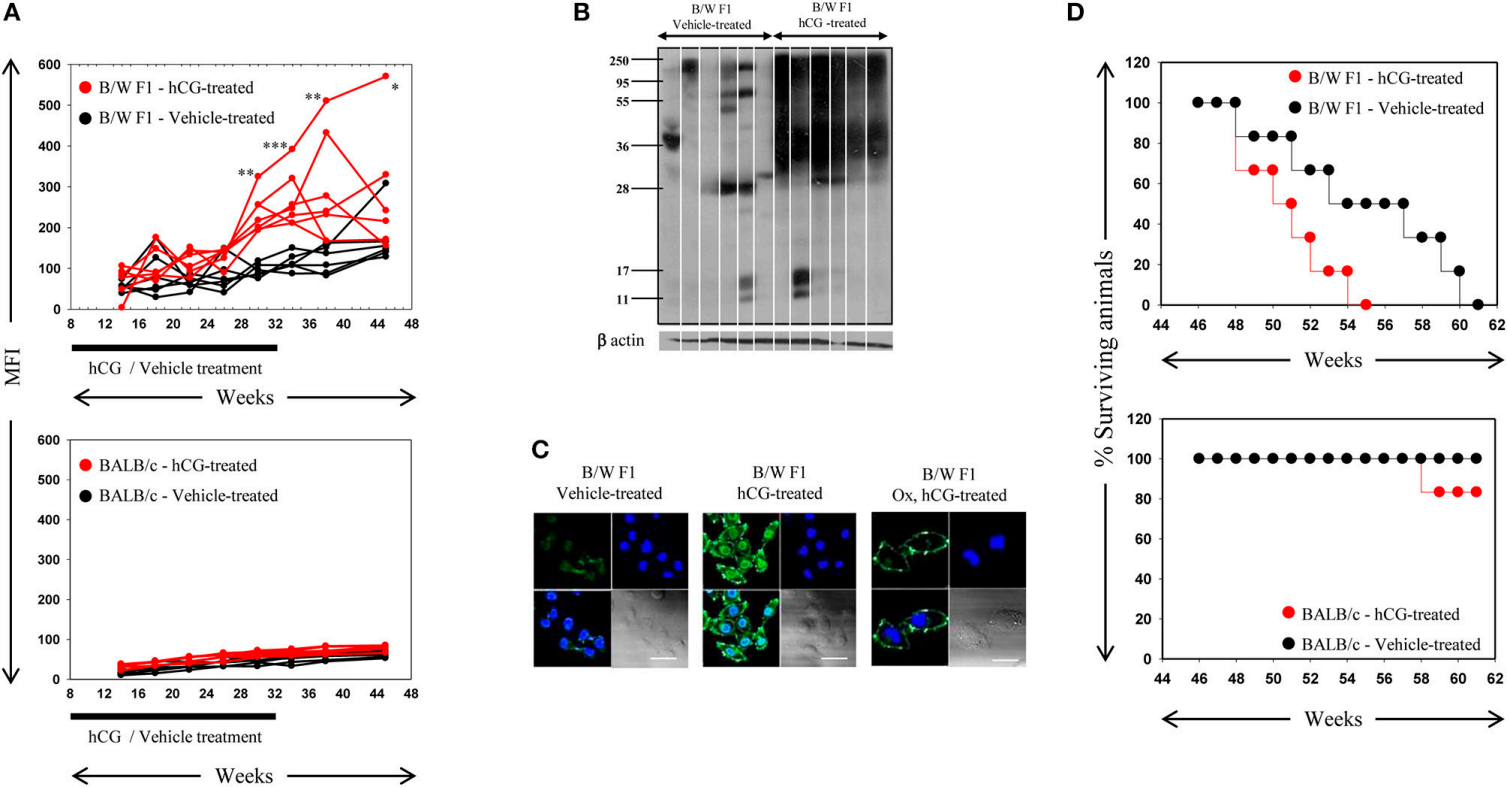
: Analysis of antibodies in the sera of B/W F1 and BALB/c mice. **(A)** Kinetics of Mean Fluorescence Intensities (MFI) upon flow cytometric analysis to assess reactivity of antibodies in the sera of vehicle-treated (black lines) and hCG-treated (red lines) B/W F1 (Top) and BALB/c (Bottom) mice toward permeabilized CCL131 cells. The duration of treatment is indicated. Each line represents an individual animal. *p < 0.05, **p < 0.01, ***p < 0.001: Vehicle-treated vs. hCG-treated B/W F1 mice at respective time-points. **(B)** Reactivity of antibodies in sera derived from individual B/W F1 (vehicle-treated and hCG-treated, Week 38) mice toward CCL131 cellular lysate by Western blot. Each lane represents an individual animal. Anti-β actin antibodies were employed to verify equivalence of loading. **(C)** Confocal immuno-fluorescence analysis of the reactivity of antibodies in pooled sera obtained from B/W F1 (vehicle-treated and hCG-treated, Week 38) mice toward CCL131 cells. Reactivity of antibodies in sera of ovairectomized (Ox), hCG-treated B/W F1 mice is also shown. Top right panels, DAPI; Top left panels, Antibody reactivity; Bottom left panels, Overlays; Bottom right panels: DIC. Bars: 20 μm. **(D)** Top panel: Kalpan-Meier analyis depicting the survival of vehicle-treated (black lines) and hCG-treated (red lines) B/W F1 mice. *p* < 0.002, vehicle-treated vs. hCG-treated mice. Bottom panel, Kalpan-Meier analyis depicting the survival of vehicle-treated (black lines) and hCG-treated (red lines) BALB/c mice.

Whether the administration of hCG in non-pregnant, lupus-prone mice could broadly mimic the effects of pregnancy in terms of elicitation of anti-self responses was assessed. Administration of hCG to B/W F1 mice induced increases in anti-self reactivity in sera (as assessed by flow cytometric analysis on permeabilized CCL131 cells), compared with vehicle-treated mice; a time-dependent increase in titres of auto-antibodies was observed (Figure 2A, Top). Administration of the hormone to BALB/c mice did not lead to similar increases in autoreactive antibody responses (Figure 2A, bottom). On Western blot, antibodies in the sera of hCG-treated B/W F1 mice demonstrated greatly increased reactivity toward CCL131 cellular lysate compared with vehicle-treated B/W F1 mice (Figure 2B); increased reactivity was also observed on lysates derived from other murine cell lines (data not shown). Confocal analysis revealed increased reactivity toward the nucleus and the cell membrane in the antibodies in the sera of hCG-treated B/W F1 mice compared to vehicle-treated mice (Figure 2C). The increased autoreactivity observed in B/W F1 mice upon the administration of hCG was associated with significantly reduced survival (Figure 2D, Top), an effect not seen BALB/c mice (Figure 2D, Bottom). The administration of hCG to ovariectomized B/W F1 mice permitted the evaluation of “direct” (subsequent to direct action on cells of the immune system) vs. “indirect” (subsequent to binding LH/CG receptors on the ovaries, for example) effects. While increased anti-self antibody responses (particularly directed toward cell-surface moieties) were observed in hCG-treated ovariectomized mice (compared with vehicle-treated mice), reactivity toward internal moieties declined compared with intact, hCG-treated mice (Figure 2C). The administration of hCG in B/W F1 mice induced an increase in titres of antibodies to several phospholipids. While ovariectomy reduced responses toward most phospholipids in hCG-treated mice, reactivity toward some phospholipids still remained above those seen in vehicle-treated B/W F1 mice, indicating that hCG-induced effects on the generation of autoreactive humoral immune responses were at least partially independent of the ovary (Supplementary Figure S1).

Alternative lupus-prone (NZM) and healthy (FVB) murine strains were employed to address whether observations of hCG-induced increase in humoral autoreactivity in a lupus milieu can be replicated. As before, flow cytometric analysis, using permeabilized CCL131 cells as targets, was carried out to initially assess the reactivity of antibodies in the sera of hCG-treated and vehicle-treated mice toward cellular moieties. Reactivity increased upon treatment with hCG in NZM mice (Supplementary Figure S2, left panel); FVB mice did not respond to the administration of hCG with a significant rise in autoreactive antibodies in sera (Supplementary Figure S2A, Right). Antibodies in sera derived from hCG-treated NZM animals were reactive toward a wide spectrum of antigens in CCL131 cell lysate on Western blot (Supplementary Figure S2B); increased reactivity was also observed on lysates derived from other murine cell lines (data not shown). Antibodies in sera derived from vehicle-treated NZM animals were much less reactive (Supplementary Figure S2B). Confocal microscopy further revealed that sera derived from hCG-treated NZM mice contained heightened levels of antibodies reactive toward cellular moieties; predominantly cytoplasmic recognition was observed. No such reactivity was observed in sera from vehicle-treated NZM mice (at the dilutions assessed) or from hCG-treated or vehicle-treated FVB mice under similar conditions (Supplementary Figure S2C).

Whether the administration of hCG results in increased autoreactivity toward common lupus-associated autoantigens was also assessed. While age-related increases were observed in antibody titres against dsDNA and RNP68 in the sera of vehicle-treated NZM as expected, the administration of hCG in NZM mice led to further increases in autoreactivity toward these moeities (Supplementary Figure S3A, Top). Autoreactivity to Protein S, Protein C, β2-glycoprotein 1 was also evaluated in hCG-treated animals, since administration of the hormone is associated with thrombotic events in humans (20, 21); while autoantibodies to β2-glycoprotein 1 registered an increase as animal aged, no such increases in levels of antibodies to Protein S, Protein C were observed. Of interest was the fact that significant increases were observed in autoantibodies directed toward Protein S and Protein C, and β2-glycoprotein 1 in hCG-treated NZM mice, compared with vehicle-treated mice (Supplementary Figure S3A, Bottom). As in NZB/W F1 mice, the administration of hCG in NZM mice induced an increase in titres of antibodies to several phospholipids as well (Supplementary Figure S3B). While the significance of these findings is discussed below, data derived from two lupus-prone murine strains suggests the potential for hCG-induced humoral autoreactivty.

Anti-hCG antibodies were not observed in the sera derived from hCG-administered lupus-prone or healthy mice (data not shown), an indication that the humoral autoreactivity observed in lupus-prone mice upon hCG administration was not simply a consequence of antibody cross-reactivity arising subsequent to the generation of anti-hCG antibody responses.

### Effect of hCG on Humoral Autoimmune Responses *ex vivo*

**Figure 3:**
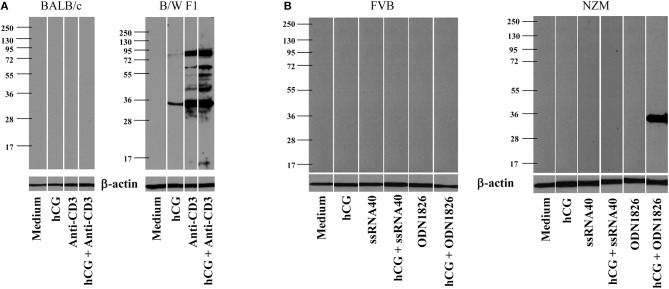
Generation of autoantibodies from splenocytes cultures. **(A)** Splenocytes isolated from BALB/c (Left) or B/W F1 (Right) mice were incubated with Medium, hCG, anti-CD3 antibodies, or hCG + anti-CD3 antibodies. Supernatants were assessed for reactivity toward apoptotic blebs by Western blot. Reactivity of anti-β actin antibodies was employed to verify equivalence of loading. **(B)** Splenocytes isolated from FVB (Left) or NZM (Right) mice were incubated with Medium, hCG, ssRNA40, hCG + ssRNA40, ODN1826, or hCG + ODN1826. Supernatants were assessed for reactivity toward apoptotic blebs by Western blot. Reactivity of anti-β actin antibodies was employed to verify equivalence of loading.

Whether hCG could synergize with a either a TCR or a TLR stimulus in a lupus milieu to induce the generation of anti-self responses *ex vivo* was assessed. In the first instance, whether the cumulative effects of hCG and anti-CD3 antibodies in splenocytes cultures would result in the enhanced generation of autoantibodies directed against components on apoptotic blebs [the primary antigenic trigger in lupus (27)] was evaluated. Interestingly, while anti-CD3 antibodies expectedly elicited autoantibodies which bound moieties in apoptotic blebs from splenocytes isolated from B/W F1 mice, the addition of hCG to splenocytes cultures too elicited a degree of humoral autoreactivity. hCG synergized with anti-CD3 antibodies to further increase the spectrum as well as titres of elicited autoreactive antibody responses in these mice. Of note was the fact splenocytes derived from BALB/c mice did not respond to these moieties with the generation of autoantibodies (Figure 3A). In the second instance, whether hCG in combination with a TLR7 ligand (ssRNA40) or a TLR9 ligand (ODN1826) would differentially elicit the generation of autoantibodies from splenocytes derived from lupus-prone mice was assessed. In NZM mice (but not in FVB mice), the combination of hCG and ODN1826 induced the generation of autoantibodies that bound a ~35 Kda moiety present in apoptotic blebs (Figure 3B). hCG, in conjunction with either a T cell receptor stimulus or a lupus-relevant TLR ligand, can therefore drive the generation of autoantibodies specifically from splenoctytes derived from lupus-prone mice.

### Effect of hCG and TLR Ligand Co-signaling on B Cell Phenotype and on Cytokine Responses

Whether hCG exerts a differential influence on the phenotype of B cells derived from lupus-prone and healthy mice was then assessed. Splenocytes derived from NZM and FVB mice were incubated with hCG alone or in combination with a TLR7 ligand (ssRNA40) or a TLR9 ligand (ODN1826), and cell surface markers on CD19^+^ cells evaluated by flow cytometry. A combination of ssRNA40 and hCG induced significantly greater increases in levels of CD40 than individual ligands alone on B cells from NZM mice; similar results were obtained when a combination of ODN1826 and hCG was employed. Such increases were significantly greater on B cells from NZM mice than on those from FVB mice. Similarly, combinations of hCG and ODN1826 significantly enhanced levels of CD86 on B cells from NZM mice over individual ligands alone, with combinatorial increases once again higher than those seen in FVB mice (Supplementary Figure S4). Interestingly, MHC 2 levels were higher on B cells in splenocyte cultures derived from FVB mice (compared to on B cells derived from NZM mice) upon individual incubation with ssRNA40 or ODN1826, a finding that may be attributable to the fact that cells derived from lupus-prone mice are sometimes stimulated only sub-optimally *in vitro*. Though ODN1826 did not induce increases MHC 2 levels on B cells in NZM mice to the extent it did in FVB mice, only in the former was there evidence of synergy when ODN1826 was added along with hCG; on the contrary, when added along with ODN1826, hCG had an ameliorating effect on MHC 2 levels on B cells derived from FVB mice (Supplementary Figure S4), in line with the hormone's previously described classical suppressive role. Further evidence of synergy between hCG and the TLR ligands in a lupus milieu was obtained when supernatants from these cultures were assessed for cytokines implicated in lupus pathogenesis. hCG, when added on its own, induced significantly higher levels of TNF-α from NZM splenocytes than from FVB splenocytes, with a combination of hCG and ssRNA40 leading to further increases over individual moieties; combinatorial increases were higher than those observed in healthy mice. Though combinations of hCG and ODN1826 resulted in greater increases in TNF-α secretion than elicited by individual moieties in both healthy and lupus-prone mice, a bias toward greater increases in the latter was apparent in this case as well. hCG elicited significantly higher levels of IL-10 from cells derived from lupus-prone mice than from healthy mice. A combination of hCG and ODN1826 elicited significantly higher levels of IL-10 than individual moieties in lupus-prone mice, an effect not observed in healthy animals. Synergistic increases in the secretion of IL-6 upon hCG and ODN1826 co-incubation were, once again, restricted to cells derived from lupus-prone mice. IFN-γ was not detected in appreciable amounts in these assays (Supplementary Figure S5). In all instances, hCG-induced effects were inhibited by the addition of anti-hCG antibodies (data not shown). hCG, when added in conjunction with TLR ligands implicated in lupus autoimmunity to splenic cell cultures derived from lupus-prone mice, therefore synergistically enhances both the levels of several co-stimulatory molecules on B cells, as well as the secretion of inflammatory, lupus-associated cytokines.

### Effect of hCG and TCR Co-Signaling on Proliferative and Cytokine Responses, and on Signaling Intermediates

**Figure 4:**
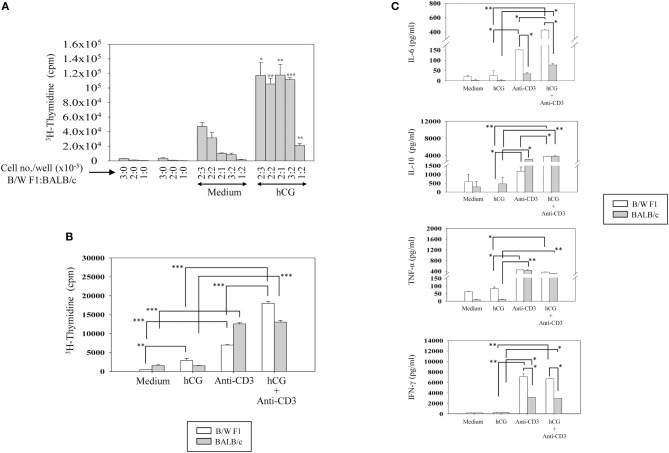
Effect of hCG on leucocyte proliferative and cytokine responses. **(A)** Effect of hCG on alloreactive proliferative responses. Spleen cells (at the indicated numbers) from B/W F1 and BALB/c mice, dispensed in varying ratios (enumerated as B/W F1:BALB/c), were co-cultured in the presence of hCG. Bars represent arithmetic means ± SEM (from four independent experiments) of ^3^H-thymidine incorporation. *p < 0.05, **p < 0.01, ***p < 0.001 vs. corresponding cultures not containing hCG. **(B)** Effect of hCG on anti-CD3 induced proliferative responses. Splenocytes isolated from BALB/c and B/W F1 mice were incubated with hCG and anti-CD3 antibodies alone and in combination. Bars represent arithmetic means ± SEM (from three independent experiments) of ^3^H-thymidine incorporation. **p < 0.01, ***p < 0.001. **(C)** Effect of hCG on cytokine seceretion by splenocytes. Splenocytes isolated from BALB/c and B/W F1 mice were stimulated with hCG and anti-CD3 antibodies alone and in combination. Bars represent arithmetic means ± SEM from three independent experiments. *p < 0.05, **p < 0.01.

Upon TCR stimulation, whether hCG differentially influences responses in lupus-prone mice was assessed. Splenocytes from BALB/c and B/W F1 mice were co-cultured at varying ratios in the presence or absence of hCG. At all cell ratios, hCG induced a significant enhancement in proliferation (Figure 4A). Whether T cells from lupus-prone mice are more sensitive to the presence of hCG than are T cells from healthy mice was assessed. hCG increased proliferative responses upon the stimulation of splenocytes isolated from B/W F1 mice with anti-CD3 antibodies; in BALB/c mice, supplementation with hCG did not enhance anti-CD3-mediated proliferation (Figure 4B). In B/W F1 mice, hCG significantly enhanced production of IL-6 and IL-10 when co-incubated with anti-CD3; for IL-6, levels attained were significantly higher for B/W F1 splenocytes upon such co-incubation than for BALB/c splenocytes. Levels of TNF-α and IFN-γ were not affected upon hCG co-incubation with anti-CD3 (Figure 4C). TGF-β, IL-8, and IL-5 were not detected in these assays (data not shown).

**Figure 5:**
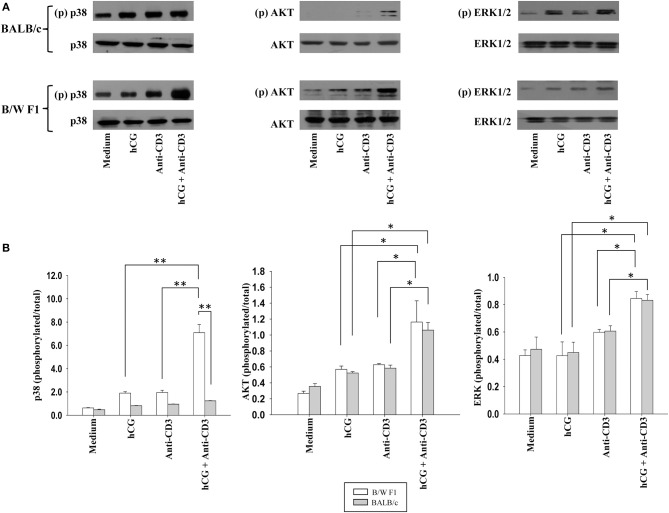
Assessment of the phosphorylation of signaling intermediates upon TCR and hCG-mediated signaling. Splenocytes from B/W F1 or BALB/c mice were incubated with Medium, hCG, anti-CD3 antibodies or hCG + anti-CD3 antibodies. **(A)** Western blots were employed to determine phosphorylated (p) and total p38, AKT, and ERK 1/2. Representative blots of 3 independent experiments are shown. **(B)** Ratios of respective phosphorylated-to-total signal band intensities as assessed by ImageJ analysis from 3 independent experiments. Bars represent arithmetic means ± SEM of band intensities. *p < 0.05, **p < 0.01.

Whether distinct signaling events could also be discerned when lupus-prone or healthy T cells were stimulated with sub-optimal doses of anti-CD3 antibodies (in the presence or absence of hCG) was then assessed. In B/W F1 splenocytes hCG, when added along with anti-CD3, led to significantly enhanced phosphorylation of p38, compared to when the two components were individually dispensed; such effects were not seen when in splenocytes derived from BALB/c mice were employed. While the combined stimulation with anti-CD3 antibodies and hCG further amplified AKT phosphorylation and of ERK1/2 phosphorylation in both BALB/c and B/W F1 mice, strain-specific effects were not observed (Figure 5). Thus, while hCG, in conjunction with TCR stimulation, enhanced levels of the phosphorylation intermediates of three important transduction pathways, signaling via p38 was particularly and specifically enhanced in lupus-prone mice. To summarize, hCG acted as a “co-stimulant” for T cells, more so in lupus-prone mice, in terms of T cell proliferative and some cytokine responses, as well as particular TCR-mediated signaling intermediates.

## Discussion

Systemic lupus erythematosus is characterized by antibody responses against a large spectrum of self-antigens (1, 28). Several autoantibody specificities are associated with pathology (29–31).

SLE mainly afflicts women of child-bearing age (32). Estrogen and prolactin have disease-promoting effects (4, 33), whereas testosterone (33) and progesterone (34) ameliorate disease. Environments skewed toward a Th2 cytokine profile [as is lupus (6)] would be expected to perpetuate autoantibody production; some reports suggest the heightened occurrence of disease flares during pregnancy (9, 35). While hCG plays a critical role in the sustenance of human pregnancy (11, 36, 37), binding sites for hCG on non-reproductive tissues (12–14, 38) are suggestive of additional functions, including immune modulation. Indeed, the administration of hCG prevents (or reduces the severity of) T cell-mediated autoimmune conditions in mice and humans (17, 18, 39, 40), a finding of some interest, given that pregnancy is associated with an amelioration of the symptoms of Th1-associated autoimmune diseases (9, 10).

While the effects of hCG on systemic autoimmune responses had not been described before the current work, increasing evidence is suggestive of an influence. While B1a B cells produce autoantibodies and proliferate in response to hCG (13), associative data link hCG with deleterious effects in systemic autoimmunity and in conditions with over-lapping pathologies. For example, elevated levels of hCG have been reported in non-pregnant SLE patients (41) and women with lupus demonstrate increased circulating levels of hCG during pregnancy (19). Levels of hCG are also elevated during pregnancy in women with preeclampsia (22, 23) a condition characterized by thrombosis, as is lupus. Further, case reports exist documenting the onset of SLE in women receiving agents (inclusive of hCG) for ovulation (42).

Given this background, this study was based on the premise that hCG may influence cells of the immune system in a differential manner in a lupus milieu. Administration of hCG in lupus-prone mice enhanced reactivity toward several cellular moieties, including known autoantigens; antibodies directed against Protein S, Protein C, and β2-glycoprotein 1, as well as various phospholipids, were significantly enhanced. In normal human pregnancies, titres of anti-Protein S and anti-Protein C antibodies are reportedly high in the first trimester and decrease in the second and third trimesters (43) mimicking the profile of circulating hCG. Interestingly, in preeclamptic pregnancies (which are characterized by enhanced hCG levels, as indicated above), while the levels of such antibodies are further elevated in the first trimester compared to levels in healthy women, equivalent time-dependent decreases are not observed (43), findings suggestive of cause and effect. Autoantibodies to Protein S have been documented in SLE and to Protein S and β2-glycoprotein 1 in SLE and anti-phospholipid syndrome (44, 45). Of additional interest is the fact that anti-phospholipid antibodies of the IgG1 isotype, shown to be associated with anti-phospholipid syndrome, were particularly enhanced upon hCG administration (data not shown). To summarize, increased levels of hCG in humans (in two thrombosis-associated patho-physiological conditions) and the administration of hCG in mice are both associated with the heightened levels of antibodies to proteins involved in the clotting cascade. The increase in anti-phospholipid reactivity upon the administration of hCG may provide leads to the study of thrombotic events surrounding OHSS, observed to occur in healthy women upon the administration of hCG (20, 21). Consistent with its ability to elicit anti-self responses when administered to lupus-prone mice, hCG induced the secretion of autoantibodies to apoptotic blebs [considered the original immunogenic stimulus in lupus (27)] when incubated with splenocytes derived from lupus-prone mice along with either anti-CD3 antibodies or with a specific TLR ligand whose relevance to lupus pathogenesis has been previously described (46–48). While on-going studies will formally demonstrate if the decrease in survival of lupus-prone mice upon the administration hCG is associated with enhanced glomerulonephritis (and if so, if these events are dependent upon the presence of the ovaries), the data are strongly indicative of the ability of hCG to enable the generation of humoral autoimmune responses in a lupus milieu.

hCG has been shown to mediate the up-modulation of CD40 and CD86 on human myeloid and lymphoid dendritic cells, inducing the enhanced proliferation of allogeneic T cells as a consequence (49). The current study demonstrated the specific stimulatory effects of hCG on the expression of CD40 and CD86 on B cells derived from lupus-prone mice, in conjunction with two TLR ligands, both of which have been implicated in lupus (46–48). Additional data indicative of a collaborative lupus-specific inflammatory stimulus, provided by the combination of hCG with either these ligands or anti-CD3 antibodies, was obtained from cytokine analysis.

While most reports indicate that hCG has a suppressive effect on T cell proliferation (50–52), trophic effects of hCG have also been reported (53). Evidence exists for the potential intersection of the hCG and TCR signaling. While hCG promotes trophoblast invasion and theca-interstitial cell proliferation by up-modulation of ERK and AKT signaling (54, 55), the production of leptin by hCG involves cross-talk between the cAMP and p38 pathways in human placental syncytiotrophoblasts (56). Upon TCR engagement, AKT and ERK are activated (57, 58) and p38 plays a central role in integrating TCR-derived signals (59). The present study has demonstrated that hCG acts as a signaling “co-stimulant,” particularly lupus-prone mice. Upon anti-CD3 stimulation, while hCG enhanced the phosphorylation of AKT and ERK in both healthy and lupus prone mice, only in the latter is an up-modulation of p38 observed. The patho-physiological consequences arising as a consequence of hCG could therefore differ, given differential signaling and differential propensity toward the production of autoantibodies in a healthy vs. lupus-prone environment.

It is probably appropriate that the effects of native hCG be compared with those of recombinant hCG in terms of the capability of inducing anti-self reactivity, and other observed responses, in a lupus milieu. Such experiments are in progress, which will also extend and expand on current observations as regards the extent to which the ovaries (which would elicit steroids in response hCG, hormones known to affect lupus onset and progression) influences differential immunological outcomes upon hCG administration. Since LH and hCG share the same receptor, such studies might also shed light on why the incidence of lupus in women decreases after menopause.

The data reveal that, possibly contrary to expectation and conventional wisdom but in line with emerging evidence, hCG can have a variety of potentially disease-promoting effects on cells of the adaptive immune system, the effects of which may become apparent in Th2-biased immune environment. While the reported influence of the hormone has immediate relevance in the context of pregnancy in systemic autoimmunity, this study also provides mechanistic leads into its possible role in pathologies not considered to have an established autoimmune etiology as such as OHSS and preeclampsia.

## Author Contributions

AD and RP designed the study, AD, RS, AB, MM, and NJ carried out the research, AD and RP wrote the manuscript.

### Conflict of Interest Statement

The authors declare that the research was conducted in the absence of any commercial or financial relationships that could be construed as a potential conflict of interest.
