# Supplementary material for: Human Chorionic Gonadotropin Influences Systemic Autoimmune Responses
Source: Front Endocrinol (Lausanne). 2018 Dec 6;9:742. doi: 10.3389/fendo.2018.00742 (PMC6291461; doi:10.3389/fendo.2018.00742)
Supplement: Supplementary file 1 [file Presentation_1.PDF]

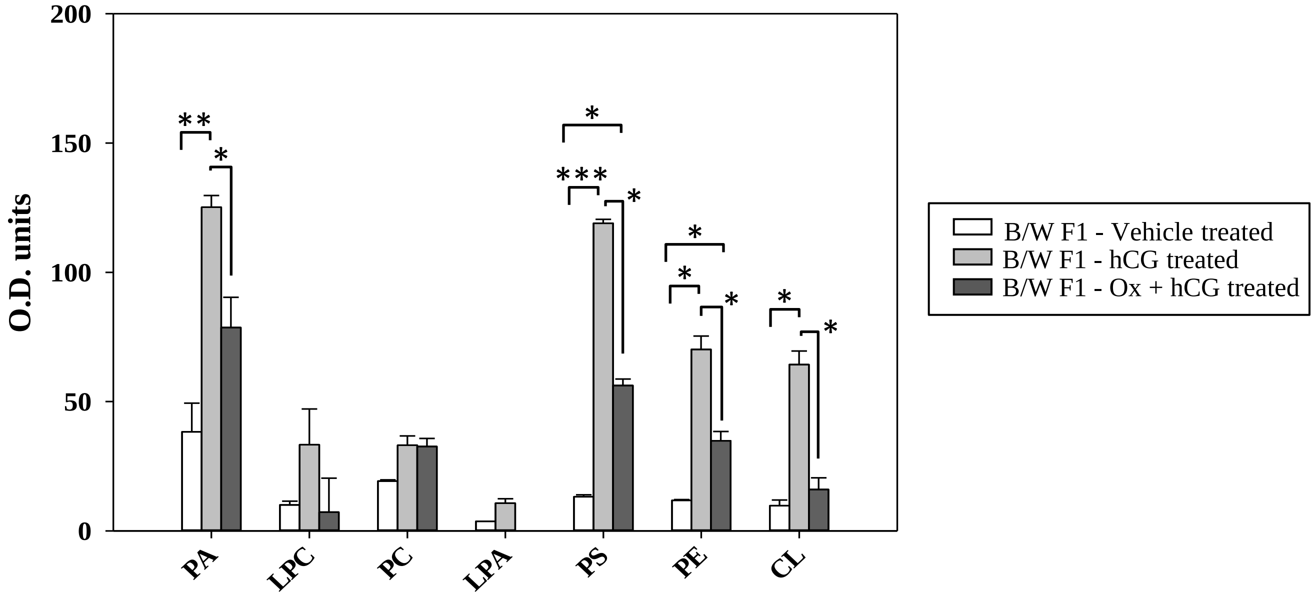

Supplementary Figure S1: Analysis of antibodies in the sera of B/W F1 mice. Anti-lipid reactivity of antibodies in pooled sera of vehicle-treated and hCG-treated B/W F1 mice (at Week 38); reactivity of antibodies in sera of ovariectomized (Ox) + hCG-treated B/W F1 mice are also shown. Bars represent arithmetic means $\pm$ SEM (from three independent experiments) of O.D. units (O.D. x serum dilution factor). *p<0.05, **p<0.01, ***p<0.001. PA: Phosphatidic Acid; LPC: Lysophosphatidylcholine; PC: Phosphatidylcholine; LPA: Lysophosphatidic acid; PS: Phosphatidylserine: PE: Phosphatidylethanolamine; CL: Cardiolipin.
