# Supplementary material for: Human Chorionic Gonadotropin Influences Systemic Autoimmune Responses
Source: Front Endocrinol (Lausanne). 2018 Dec 6;9:742. doi: 10.3389/fendo.2018.00742 (PMC6291461; doi:10.3389/fendo.2018.00742)
Supplement: Supplementary file 2 [file Presentation_2.PDF]

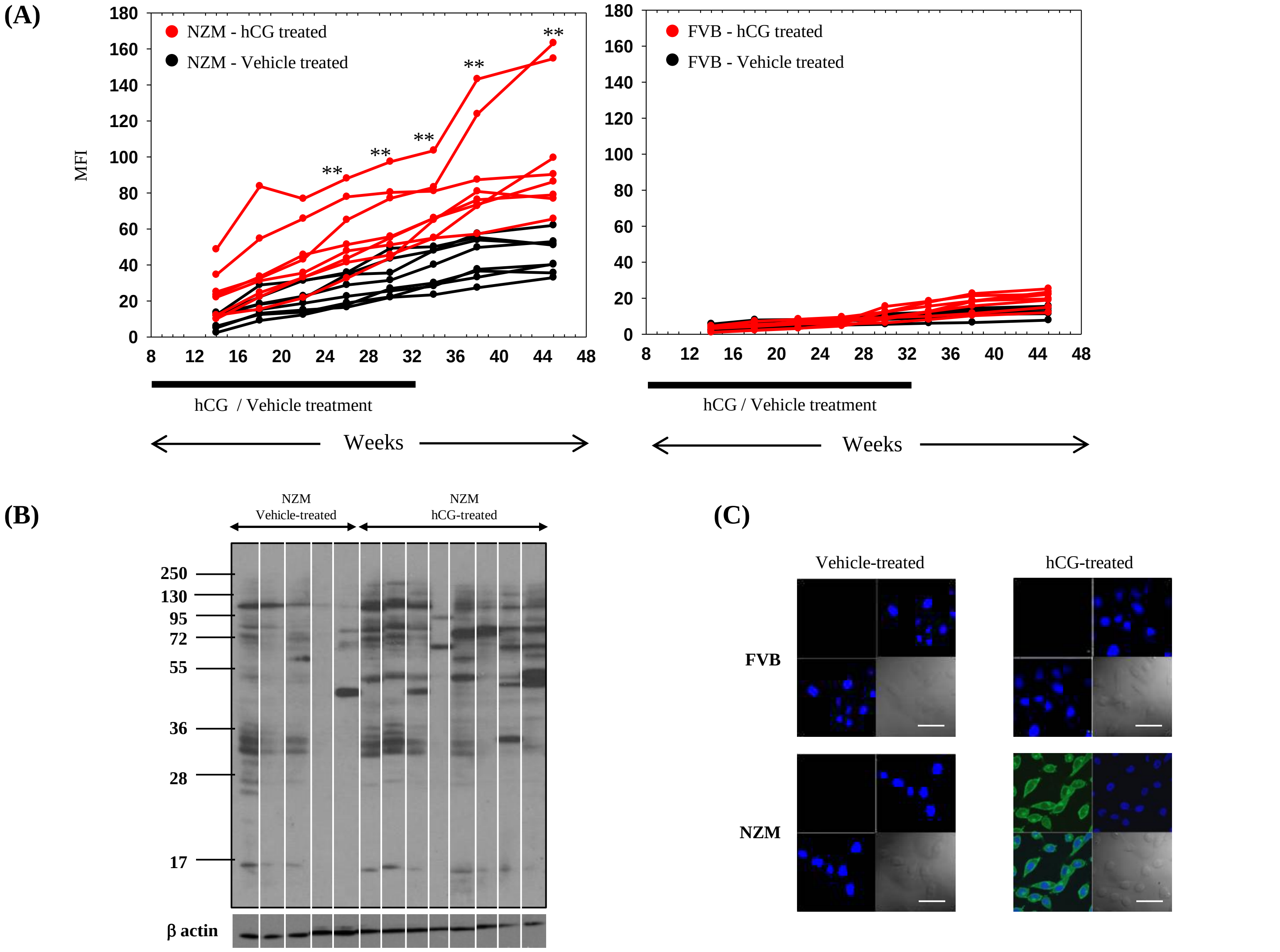

Supplementary Figure S2: Analysis of antibodies in the sera of NZM and FVB mice. (A) Kinetics of Mean Fluorescence Intensities (MFI) upon flow cytometric analysis to assess reactivity of antibodies in the sera of vehicle-treated (black lines) and hCG-treated (red lines) NZM (left panel) and FVB (right panel) mice towards permeabilized CCL131 cells. The duration of treatment is indicated. Each line represents an individual animal. *p<0.05, **p<0.01: Vehicle-treated versus hCG-treated B/W F1 mice at respective time-points. (B) Reactivity of antibodies in sera derived from individual NZM (vehicle-treated and hCG-treated, Week 38) mice towards CCL131 cell lysate on Western blot. Each lane represents an individual animal. Anti-β actin antibodies were employed to verify equivalence of loading. (C) Confocal immuno-fluorescence analysis of the reactivity of antibodies in pooled sera obtained from FVB and NZM (vehicle-treated and hCG-treated, Week 38) mice towards CCL131 cells. Top right panels: DAPI; Top left panels: Antibody reactivity; Bottom left panel: Overlays; Bottom right panels: DIC. Bars: 20 μm.
