# Supplementary material for: Human Chorionic Gonadotropin Influences Systemic Autoimmune Responses
Source: Front Endocrinol (Lausanne). 2018 Dec 6;9:742. doi: 10.3389/fendo.2018.00742 (PMC6291461; doi:10.3389/fendo.2018.00742)
Supplement: Supplementary file 3 [file Presentation_3.PDF]

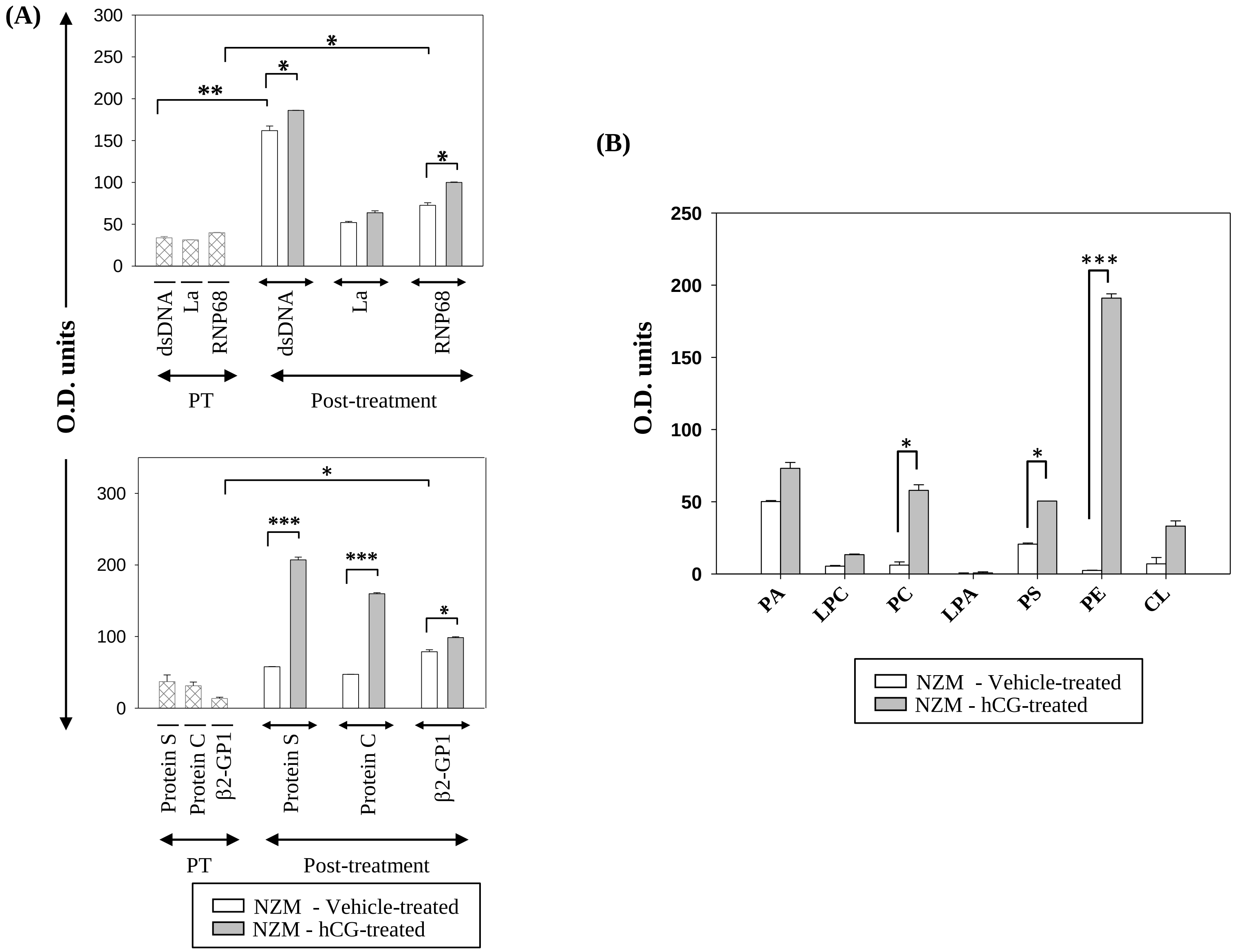

Supplementary Figure S3: Reactivity of antibodies in pooled sera from NZM (vehicle-treated and hCG-treated, Week 38) mice towards conventional autoantigens and clotting factors. (A) Reactivity towards dsDNA, ribonucleoprotein autoantigens (top panel) and clotting factors (bottom panel). Bars represent arithmetic means ± SEM (from four independent experiments) of O.D. units (O.D. x dilution factor). PT: Pre-treatment. (B) Reactivity towards phospholipids. Bars represents arithmetic means ± SEM (from three independent experiments) of O.D. units (O.D. x serum dilution factor). *p<0.05, **p<.001, ***p<0.001. PA: Phosphatidic Acid; LPC: Lysophosphatidylcholine; PC: Phosphatidylcholine; LPA: Lysophosphatidic acid; PS: Phosphatidylserine: PE: Phosphatidylethanolamine; CL: Cardiolipin.
