# Supplementary material for: Human Chorionic Gonadotropin Influences Systemic Autoimmune Responses
Source: Front Endocrinol (Lausanne). 2018 Dec 6;9:742. doi: 10.3389/fendo.2018.00742 (PMC6291461; doi:10.3389/fendo.2018.00742)
Supplement: Supplementary file 5 [file Presentation_5.PDF]

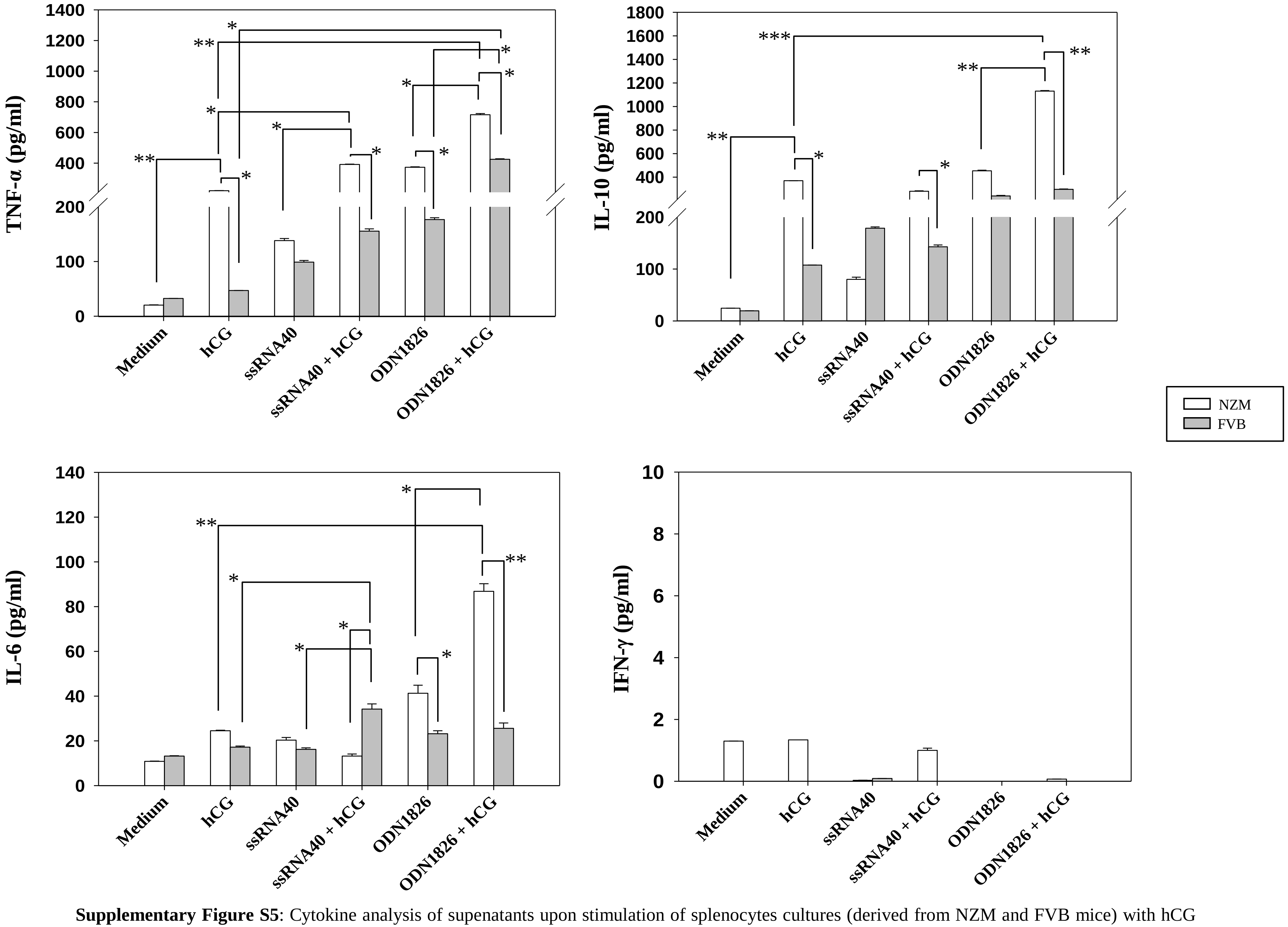

Supplementary Figure S5: Cytokine analysis of supenatants upon stimulation of splenocytes cultures (derived from NZM and FVB mice) with hCG and/or TLR ligands (ssRNA40 and ODN1826). Arithmetic means ± SEM are shown. *p<0.05, **p<0.01, ***p<0.001.
